# Investigating the widespread introduction of a tropical marine fouling species

**DOI:** 10.1002/ece3.2065

**Published:** 2016-03-11

**Authors:** Elizabeth A. Sheets, C. Sarah Cohen, Gregory M. Ruiz, Rosana M. da Rocha

**Affiliations:** ^1^ Romberg Tiburon Center for Environmental StudiesBiology DepartmentSan Francisco State University3150 Paradise DriveTiburonCalifornia94920; ^2^ Smithsonian Environmental Research Center627 Contees Wharf Rd.EdgewaterMaryland21037; ^3^ Departamento de ZoologiaUniversidade Federal do ParanáCP 1902081531‐980CuritibaBrazil

**Keywords:** Ascidian, biological introduction, *Botrylloides nigrum*, chimerism, Panama Canal, population genetics

## Abstract

Little is known about the number and rate of introductions into terrestrial and marine tropical regions, and if introduction patterns and processes differ from temperate latitudes. Botryllid ascidians (marine invertebrate chordates) are an interesting group to study such introduction differences because several congeners have established populations across latitudes. While temperate botryllid invasions have been repeatedly highlighted, the global spread of tropical *Botrylloides nigrum* (Herdman, 1886) has been largely ignored. We sampled *B. nigrum* from 16 worldwide warm water locations, including around the Panama Canal, one of the largest shipping hubs in the world and a possible introduction corridor. Using mitochondrial (COI) and nuclear (ANT) markers, we discovered a single species with low genetic divergence and diversity that has established in the Atlantic, Pacific, Indo‐Pacific, and Mediterranean Oceans. The Atlantic Ocean contained the highest diversity and multilocus theta estimates and may be a source for introductions to other regions. A high frequency of one mitochondrial haplotype was detected in Pacific populations that may represent a recent introduction in this region. In comparison to temperate relatives, *B. nigrum* displayed lower (but similar to temperate *Botrylloides violaceus*) genetic divergence and diversity at both loci that may represent a more recent global spread or differences in introduction pressures in tropical regions. Additionally, chimeras (genetically distinct individuals sharing a single body) were detected in three populations by the mitochondrial locus and validated using cloning, and these individuals contained new haplotype diversity not detected in any other colonies.

## Introduction

Marine biological introductions have increased at an exponential rate of detection globally over the last two centuries in many temperate areas (Ruiz et al. 2000; Streftaris et al. 2005). Yet little is known about the number of introduced species and their rates of introduction in tropical marine regions (Ruiz et al. 2009). Fewer alien species have been reported in tropical compared to temperate latitudes in both marine and terrestrial ecosystems (Rejmanek 1996; Sax 2001; Ruiz et al. 2009). However, this may be because of poor systematic and historical data in tropical regions (Carlton 2009). Extensive surveys in the Hawaiian Islands and Guam have revealed high numbers of marine alien species (Paulay et al. 2002; Smith et al. 2002; Lambert 2003; Coles et al. 2006; Carlton and Eldredge 2009). Differences in available taxonomic information, biogeographic knowledge, and survey effort result in uneven data quality and quantity that can make comparisons of introduction patterns between tropical and temperate regions especially challenging (Hewitt 2002).

Many introduction events also go unrecognized because an alien is mistaken for a native or previously reported introduced species (Geller et al. 2010). Additionally, species complexes may also occur where distinct evolutionary lineages display differing distributions (Fehlauer‐Ale et al. 2014). Failure to identify these cryptic introductions may underestimate their frequency and misidentify or overlook routes and vectors of introduction to new areas. Advances in molecular tools and population genetic theory can address these gaps in information (Roman and Darling 2007; Geller et al. 2010; Rius et al. 2014).

Ascidians are common marine invaders, generally found fouling boat hulls, artificial structures, and aquaculture, and display broad environmental tolerances, fast growth rates, and may be subject to limited predation (Lambert 2007). They are often difficult to distinguish morphologically and detection of new and existing introductions can be challenging without the use of genetic methods (Stefaniak et al. 2009; Zhan et al. 2010; Cohen et al. 2011; Teske et al. 2011; Bock et al. 2012; Vandepas et al. 2015). These sessile organisms have a short, geographically limited planktonic larval stage and their global spread has been attributed to boat traffic and aquaculture translocation (Cohen 1998; Ruiz et al. 2000). When introduced to new regions, ascidians are capable of altering native habitats (Castilla et al. 2004) and species composition (Blum et al. 2007), blanketing acres of natural habitat (Bullard et al. 2007), as well as negatively impacting aquaculture (McKindsey et al. 2007; Carman et al. 2010) and fisheries (Valentine et al. 2007).

In the botryllid clade, two widely introduced temperate species, *Botryllus schlosseri* and *Botrylloides violaceus*, are important models for invasion biology: their population structure, environmental tolerances, and invasion impacts have been investigated in regions worldwide (Brunetti et al. 1984; Carver et al. 2006; McCarthy et al. 2007; Epelbaum et al. 2009; Carman et al. 2010). This group also contains interesting life history characters that may contribute to their successful global spread, such as whole body regeneration from small vascular fragments (Davis 1988; Rinkevich et al. 1995) and fusing with very closely related kin to form chimeras (Cohen et al. 1998; Ben‐Shlomo et al. 2007). The role of fusion in the field is still poorly understood, but studies have suggested various benefits and trade‐offs (Grosberg 1988; Chadwick‐Furman and Weissman 2003; Rinkevich and Yankelevich 2004). Chimeras may occur at high rates (fusion has previously been detected in 75% of recruits) in natural botryllid populations (Westerman et al. 2009) and have been identified using variable genetic markers in botryllids and other ascidian families (Ben‐Shlomo et al. 2007; Stefaniak et al. 2009; Pérez‐Portela et al. 2012). Despite the substantive research on the global introductions of temperate botryllids, widespread introductions of tropical relatives have been largely ignored. The global distribution of tropical *Botrylloides nigrum* provides an interesting opportunity to compare introduction patterns of phylogenetically close species with similar introduction vectors in tropical versus temperate locations.

*Botrylloides nigrum* (Herdman, 1886), first described in Bermuda, is currently reported in tropical and warm water regions across the globe. This broad and disjunct geographic pattern may include multiple unrecognized cryptic species, as botryllid species are particularly difficult to distinguish morphologically and many regions in the tropics have poor historical biodiversity records. The global distribution of *Botryllus schlosseri* shows both spatially restricted and broadly introduced evolutionary lineages (Bock et al. 2012; Yund et al. 2015), while the *Botrylloides violaceus* global introduction currently reveals a single lineage (Lejeusne et al. 2011). *Botrylloides nigrum* is also currently classified as cryptogenic across its range, such that the native versus non‐native distribution is poorly understood (Rocha 2009; Carman et al. 2011). Both *Botryllus schlosseri* and *Botrylloides violaceus* have retained genetic signals between their native versus introduced ranges (Lopez‐Legentil et al. 2006; Lejeusne et al. 2011). The warm West Atlantic is the hypothesized native source of *B. nigrum*, where it is frequently reported from northern Florida to southern Brazil (Carlton and Ruckelshaus 1997; Rocha and Bonnet 2009).

The Panama Canal, one of the most heavily visited ports in the world (Kaluza et al. 2010), has been identified as a potential tropical hotspot for marine introductions, but little is known about how the canal contributes to the biotic exchange of many taxa between the Atlantic and Pacific Oceans (Cohen 1972, 2006; Ruiz et al. 2006). Ascidian introductions through the Panama Canal are suspected: surveys have reported at least seven species that are present at both the Atlantic and Pacific entrances, including *B. nigrum* (Cohen 2006; Carman et al. 2011). Sampling populations of *B. nigrum* at each entrance to the canal and at greater distances may help us better understand the role of the Panama Canal in introductions of low‐dispersing fouling species between oceans. However, the greater distribution of *B. nigrum* outside the Panama Canal area on the Pacific Coast of the Americas is unknown. Gathering baseline data of populations around the canal is critical for understanding how shipping may affect introductions over time, particularly as the future extension of the canal is estimated to dramatically increase the number of transits between the Atlantic and Pacific Oceans (Reagan 2007).

In this study, we investigated the global population structure and introduction history of the sessile marine invertebrate *Botrylloides nigrum*. We obtained a dataset of mitochondrial and nuclear sequences from populations across its global distribution to investigate: (1) Does the environmentally broad and geographically disjunct distribution of this species reveal cryptic diversity or the global introduction of a single species? (2) What phylogeographic patterns characterize this understudied tropical introduced species? (3) And how do these patterns compare to introductions of temperate relatives?

## Materials and Methods

### Sampling and genetic methods

Samples identified morphologically as *Botrylloides nigrum* were collected from 16 locations (Table 1). Sampling in this study of the West Atlantic and the Pacific Coast of the Panama Canal covered a large portion of the previously reported distribution of *B. nigrum*, excluding Guam, Somalia, West Africa, the Indian Ocean, and the Red Sea (Millar 1955; Fishelson 1971; Millar 1988; Lambert 2003; Shenkar 2012). Discussions with global colleagues allowed us to target and sample unconfirmed botryllid species found in marinas in Hawaii, both coasts of Mexico, a buoy in Singapore, and a suspected misidentified botryllid on the Mediterranean coast of Israel. Samples were generally collected at least 1 m apart to avoid sampling clones or close relatives among this species, as it has extremely limited natural dispersal. All samples were preserved and stored for genetic analysis in 95% ethanol.

**Table 1:** Population code, population location, geographic region, and average annual sea temperature range for populations of *Botrylloides nigrum* investigated in this study. For each location, the average coldest and warmest temperatures of the year are shown as the annual temperature range

Code	Site	Basin	Annual temperature range (°C)
IR	Indian River, Florida, USA	Northwest Atlantic	22.4–29.3
TB	Tampa Bay, Florida, USA	Gulf of Mexico	18.2–30.2
TX	South Padre Island, Texas, USA	Gulf of Mexico	19.2–29.5
VR	Veracruz, Mexico	Gulf of Mexico	22.9–29.8
BA	Bahamas	North Caribbean	23.3–30.1
BZ	Twin Cayes/South Water, Belize	North Caribbean	26.4–29.4
PR	San Juan, Puerto Rico	North Caribbean	24.9–29.2
VZ	Margarita Island, Venezuela	South Caribbean	27.1–29.4
BOC	Bocas, Panama	South Caribbean	27.6–29.2
COL	Colon, Panama	South Caribbean	26.7–29.1
BR	Florianópolis/Ilhabela, Brazil	Southwest Atlantic	21.2–26.5
MAZ	Mazatlan, Nayarit, Mexico	Eastern Pacific	23.9–30.3
PA	Panama City, Panama	Eastern Pacific	26.2–28.8
HI	Oahu, Hawaii, USA	Central Pacific	24.3–27.0
SGP	Singapore	Indo‐Pacific	27.6–30.1
IS	Haifa, Israel	Mediterranean	16.4–29.3

Temperate data from Seatemperature.org was collected in 2012–2013 and was taken from closest location (within 150 km) to our genetic sampling site.

Genomic DNA was extracted from individuals by dissecting a 0.5 cm^2^ piece of tissue containing zooids from each colony and using a NucleoSpin Tissue kit (Machery‐Nagel). Genetic diversity was assessed using the mitochondrial cytochrome *c* oxidase I (COI) gene and nuclear adenine nucleotide translocate (ANT) gene because these markers have proven to be useful for identifying species diversity and phylogeographic patterns in invertebrates, including ascidians (Avise et al. 1987; Pineda et al. 2011). We used primers *Tun_forward* (Stefaniak et al. 2009) and *HCO2198* (Folmer et al. 1994) to amplify COI, and primers *ANTf1* (Jarman et al. 2002) and *ANTr_Splic* (Pineda et al. 2011) to amplify ANT. Amplification for each locus was carried out in a 25 *μ*L reaction mixture with 1.5 mmol/L MgCl2, 0.2 mmol/L dNTPs, 1× PE buffer, 0.5 *μ*mol/L of each primer, 0.4 mg bovine serum albumin, one unit *Taq* (New England Biolabs, Ipswich, MA), and 20–300 ng of template DNA. Initial denaturing at 95°C for 3 min was followed by 35 amplification cycles (denaturing at 95°C for 45 sec, annealing at 44–54°C for 45 sec, and extension at 72°C for 1 min) and a final extension at 72°C for 10 min. PCR products were cleaned with ExoSAP‐IT^®^ (Affymetrix, Santa Clara, CA) and sequenced using 1/8 BigDyeTerminator v3.1 (Applied Biosystems, Foster City, CA) cycle sequencing reactions and an ABI 3130 genetic analyzer (Applied Biosystems) at the Romberg Tiburon Center for Environmental Studies.

COI and ANT sequences were trimmed and aligned visually in SEQUENCHER v4.8 (Gene Codes Corporation, Ann Arbor, MI). To confirm species identity of collected samples, the COI marker was used to amplify specimens of *B. nigrum* collected from Panama and Brazil that were identified using the following diagnostic taxonomic features: a characteristic color pattern of living colonies that includes an orange or white horseshoe shaped line linking the siphons against a dark violet or black tunic, the cardiac end of the stomach is generally wide and truncated with the ends of the folds forming conspicuous and prominent rounded bulbous projections, a short cecum enlarged toward the blind distal end that was often curved but only slightly, and usually one oocyte at each side between the testis and the gut (Van Name 1945; Monniot 1983).

Samples amplified at the nuclear ANT locus were directly sequenced and secondary peaks from heterozygous individuals were called by eye in SEQUENCHER. Alleles were determined using PHASE v2.1 (Stephens et al. 2001; Stephens and Scheet 2005) and unique haplotypes were verified by cloning using the pGEM T Vector System II kit (Promega). We tested for recombination in the ANT gene using RPD4 (Martin et al. 2010). COI and ANT alleles that were detected in only one individual were amplified and sequenced a second time to confirm single nucleotide polymorphisms. For specimens with COI sequences showing multiple peaks in chromatograms, we considered the possibility of contamination, pseudogenes, or chimerism. To evaluate these possibilities, genomic DNA was extracted from each colony an additional two times by dissecting a single zooid from opposite ends of the colony. Samples showing heterogeneity in all extractions were cloned (using the same methods as above) to identify haplotypes. These samples were included in our dataset (see Results: Heteroplasmy in COI).

### Population analysis

Number of alleles (Nh), gene diversity *(Hd),* and nucleotide diversity *(π)* were estimated in ARLEQUIN v3.5 (Excoffier and Lischer 2010). Three of the 16 populations in this study had very low sampling (Bahamas; Colon, Panama; Singapore) and were excluded from population analyses, but were included when investigating regional basins and oceans. Allelic richness was rarefied across populations (COI: *n *=* *7, ANT: *n *=* *14) and ocean basins (COI: *n *=* *30, ANT: *n *=* *60) in CONTRIB v1.02 (Petit et al. 1998). The inbreeding coefficient *F*_IS_ along with expected and observed heterozygosity of ANT were estimated in GENETIX v4 (Belkhir et al. 2004). *F*_IS_ values were considered significant if 90% or more of the values sampled after 1000 permutations were greater than or less than zero.

The online program FINDMODEL determined that the Tamura‐Nei and Jukes‐Cantor models were the best DNA substitution models for COI and ANT respectively. Pairwise *F*_ST_ and Φ_ST_ (incorporating the Tamura‐Nei and Jukes‐Cantor models) comparisons for 13 populations were estimated in ARLEQUIN. For these two estimates, the online SISA program (Uitenbroe 1997) estimated a Bonferroni correction *P*‐value of <0.004 that was implemented in ARLEQUIN to correct for multiple pairwise comparisons. Population structure was examined in ARLEQUIN by performing a hierarchical analysis of molecular variance (AMOVA) with 10,000 permutations to test significance. Three separate AMOVA analyses were conducted by grouping sequences at different geographic scales, including (1) no grouping of populations, (2) grouping by local basin, and (3) grouping by ocean (see Table 1 for location information). For each locus, a correlation of genetic distance and the log of geographic distance was tested among all samples using a Mantel test with 10,000 permutations in the program GENALEX v6.5 (Peakall and Smouse 2006). To estimate if non‐neutral selective pressures or past changes in population size affected the COI or ANT loci, four neutrality statistics were calculated in DNASP v5.10 and their significance values were estimated using coalescent simulations with 5,000 replicates: Tajima's D (Tajima 1989), Fu's Fs (Fu 1997), Fu and Li's F & D (Fu and Li 1993), and Ramos‐Onsins and Roza's statistic (Ramos‐Onsins and Rozas 2002).

To assess gene flow among ocean basins, we used the software package MIGRATE v3.5 (Beerli, 2013) to estimate the effective number of migrants per generation (*N*_M_ = *θm*) entering and leaving each ocean basin. Since mitochondrial and nuclear organelles have different evolutionary histories, separate analyses for COI and ANT were conducted. To avoid effects of differences in sample size on gene flow estimates, the program randomly subsampled 30 COI alleles and 60 ANT alleles from each ocean population. The transition to transversion ratio of each locus was calculated in the program MEGA v5.2 (Tamura et al. 2011) for use in these analyses (COI = 13.88, ANT = 0.5). A uniform prior migration rate was set with a minimum 0 and a maximum of 10,000. Analysis involved 10 replicates of one long chain that recorded 1,000,000 steps and a burn‐in of 500,000. To better sample the posterior space, a static heating scheme was used and consisted of eight chains set with default temperatures. To confirm convergence of estimates, five analyses with varying starting seeds were conducted. An average of *θ* and *m* mean estimates from each analysis were used to calculate *N*_M_ (*θm* for COI, 0.25*θm* for ANT) between each ocean basin.

### Phylogenetic analysis

For both loci, the following analyses were conducted to investigate relationships among alleles. Minimum spanning networks were estimated in ARLEQUIN and imported to HAPSTAR, a program that generates unbiased visual layouts of allelic relationships (Teacher and Griffiths 2010). Synonymous and nonsynonymous substitutions were detected in MEGA. Phylogenetic relationships were estimated using both Maximum Likelihood and Bayesian inference, conducted in MEGA and MRBAYES v3.2 (Ronquist et al. 2012) respectively. For COI phylogenetic analysis, a previously published sequence of *Botrylloides leachi* (Genbank Accession FJ528644) was used as an outgroup since it is a closely related congener (Griggio et al. 2014). Since there is no published sequence of a congener for the same ANT locus region, we amplified a *Botrylloides violaceus* specimen collected from San Francisco Bay, CA, USA, and confirmed its species identity using the COI locus.

## Results

### Mitochondrial cytochrome c oxidase subunit I (COI) gene

**Figure 1:**
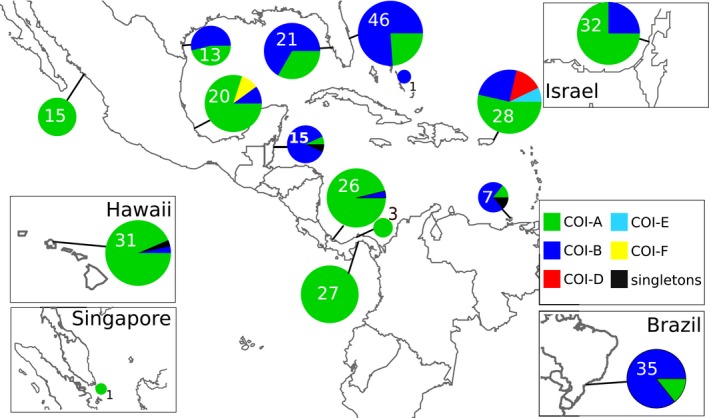
Map showing sampling sites of *Botrylloides nigrum* and COI haplotypes. Circles represent populations, the size of circles and the numbers within circles represents sample size, and colors represent alleles.

In our final set of 321 samples from 16 locations, a 527 nt fragment of COI was used for analysis. This study detected eight alleles with 10 (2%) polymorphic sites, where eight of these sites were synonymous and two sites were nonsynonymous substitutions. Both nonsynonymous substitutions were only found in chimeric individuals (see Heteroplasmy in COI). We detected eight total haplotypes: two (COI‐A and COI‐B) were found globally (in at least one location in all sampled oceans) and the remaining six were private (Tables 2 and S1, Fig. 1). One global haplotype (COI‐B) had a low frequency (*n *=* *1) in one location, Hawaii. The Atlantic Ocean contained the greatest number of haplotypes (seven in 215 individuals) compared to the Pacific (three in 74 individuals) and Mediterranean (two in 32 individuals).

**Table 2:** Genetic diversity measures of *Botrylloides nigrum* populations for two loci (COI and ANT)

Site^a^	COI					ANT							
	N	Ar	Hd ± SD	*π* ± SD	Nh (p)	N	Ar	Hd ± SD	*π* ± SD	Nh (p)	*F*_IS_	*H*_exp_	*H*_obs_ b
Atlantic	215	2.328	0.540 ± 0.016	0.004 ± 0.002	7 (5)	206	4.194	0.684 ± 0.016	0.009 ± 0.006	10 (3)	**0.193**	0.688	0.557
IR	46	0.874	0.372 ± 0.067	0.003 ± 0.002	2	44	3.203	0.618 ± 0.049	0.008 ± 0.005	6 (2)	0.006	0.611	0.614
TB	21	0.97	0.467 ± 0.075	0.004 ± 0.002	2	22	2.901	0.568 ± 0.077	0.008 ± 0.005	5	**0.525**	0.555	0.273
TX	13	0.999	0.513 ± 0.082	0.004 ± 0.003	2	13	2.538	0.717 ± 0.034	0.010 ± 0.006	4	**−0.189**	0.690	0.846
VR	20	1.179	0.358 ± 0.127	0.003 ± 0.003	3 (1)	17	2.794	0.720 ± 0.037	0.014 ± 0.008	4	**0.271**	0.699	0.529
BA	1	–	–	–	1	1	–	–	–	1	–	–	–
BZ	15	0.933	0.257 ± 0.142	0.002 ± 0.002	3 (1)	15	1.415	0.287 ± 0.092	0.001 ± 0.001	2	0.311	0.278	0.200
PR	28	2.052	0.648 ± 0.071	0.005 ± 0.003	4 (2)	24	3.204	0.721 ± 0.035	0.011 ± 0.006	6 (1)	0.077	0.706	0.667
VZ	7	2	0.524 ± 0.209	0.003 ± 0.002	3 (1)	7	1	0.440 ± 0.112	0.002 ± 0.002	2	**1.000**	0.408	0.000
BOC	26	0.269	0.077 ± 0.079	0.001 ± 0.001	2	26	2.919	0.698 ± 0.040	0.009 ± 0.005	6 (1)	**0.184**	0.691	0.577
COL	3	–	0.000 ± 0.000	0.000 ± 0.000	1	3	–	0.333 ± 0.215	0.008 ± 0.005	2	–	–	–
BR	35	0.697	0.252 ± 0.086	0.002 ± 0.002	2	34	3.07	0.716 ± 0.033	0.011 ± 0.007	5 (1)	**−**0.069	0.705	0.765
Pacific	74	0.865	0.054 ± 0.037	0.000 ± 0.000	3 (1)	78	4.098	0.567 ± 0.041	0.008 ± 0.005	7 (1)	**−**0.060	0.555	0.592
MAZ	15	0	0.000 ± 0.000	0.000 ± 0.000	1	15	2.315	0.522 ± 0.091	0.001 ± 0.006	4	0.240	0.504	0.400
PA	27	0	0.000 ± 0.000	0.000 ± 0.000	1	30	2.326	0.512 ± 0.064	0.010 ± 0.006	5 (1)	**−**0.108	0.486	0.546
HI	31	0.452	0.127 ± 0.080	0.001 ± 0.001	3 (1)	32	2.524	0.602 ± 0.052	0.006 ± 0.004	5	**−0.251**	0.592	0.750
SGP	1	–	–	–	1	1	–	1.000 ± 0.500	0.026 ± 0.013	2	–	–	–
Medit/IS	32	1	0.387 ± 0.078	0.003 ± 0.002	2	33	4	0.723 ± 0.024	0.013 ± 0.008	5 (1)	**0.167**	0.715	0.606
Total	321	3.023	0.518 ± 0.015	0.004 ± 0.002	8	317	5.422	0.674 ± 0.014	0.010 ± 0.006	13	**0.148**	0.673	0.575

a Site abbreviations as shown in Table 1.

b Summary statistics included are: N, sample size; Ar, allelic richness with rarefaction (COI: [30] ocean, [7] population; ANT: [60] ocean, [14] population); Hd, gene diversity; *π*, nucleotide diversity; Nh: number of haplotypes, including number of private haplotypes in parentheses; *F*_IS_, Weir & Cockerham (1984) inbreeding coefficient; *H*_exp_, expected heterozygosity; and *H*_obs_ observed heterozygosity. Bolded values represent significant *F*_IS_ values (*P *<* *0.05).

**Figure 2:**
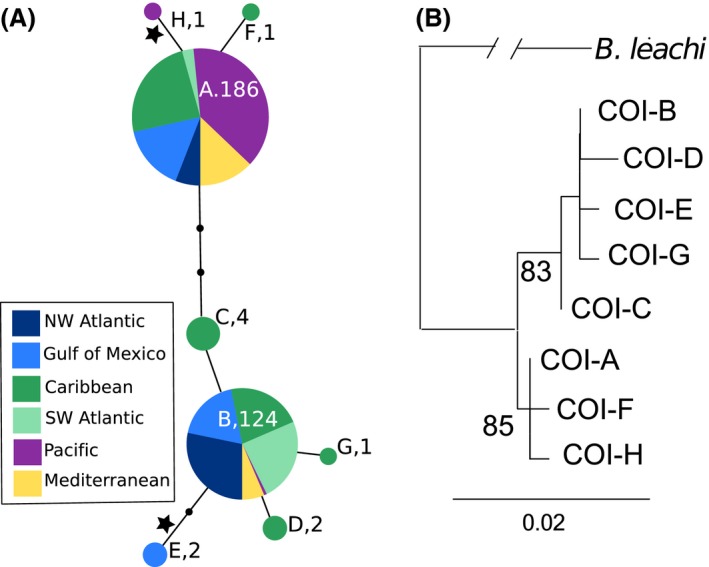
(A) Minimum spanning network for the COI locus. Circles are labeled with the haplotype name and number of individuals with that haplotype, size differences of circles reflect frequency, colors represent regions where the haplotype was found, and stars represent nonsynonymous mutations. (B) Phylogeny of 527‐bp region of the COI locus using Maximum likelihood analysis and the Tamura and Nei model. Nodes with bootstrap support values >70 are shown.

Total gene diversity was 0.5176 (±0.0145 SD) and total nucleotide diversity was 0.0038 (±0.0024 SD). Gene and nucleotide diversity varied considerably within and between regions (Table 2). For example, the Caribbean contained populations with the highest diversity at COI across measures; however, gene (0.0769 ± 0.0697 SD) and nucleotide (0.00059 ± 0.0007 SD) diversity in Bocas del Toro, Panama were considerably lower than other populations in the Caribbean. Pacific populations had substantially lower diversity: for example, gene and nucleotide diversity were zero in Mazatlan (*n *=* *15) and Panama (*n *=* *27). Puerto Rico had the highest overall gene (0.6481 ± 0.071 SD) and nucleotide (0.0054 ± 0.0032 SD) diversity of any population. The highest allelic richness values (populations rarefied to a sample size of seven) were found in Puerto Rico (2.052) and Venezuela (2.000); the Pacific populations showed the lowest allelic richness (ocean comparisons were rarefied to a sample size of 32). When comparing oceans, the Atlantic had the highest allelic richness (2.328) followed by the Mediterranean (1.000) and Pacific (0.865). Overall mean divergence was 0.4% among COI haplotypes. Maximum likelihood analysis revealed two groupings of haplotypes (Fig. 2B).

Results from AMOVA showed significant genetic structure among groups when populations were pooled into the Atlantic, Pacific, and Mediterranean Oceans (Table 3). However, no significant structure was detected when populations were pooled into regional basins (i.e. North Atlantic, Gulf of Mexico, Caribbean, South Atlantic, Pacific, and Mediterranean). When populations were pooled by ocean or basin, within population diversity was higher than diversity among populations within groups (*P* < 0.001 for both).

**Table 3:** Analysis of molecular variance (AMOVA) of populations of *Botrylloides nigrum* at each locus. Asterisks represent significant values (*P <* *0.05 ** *P *<* *0.001)

Source of variation	df	Sum of squares	Variance components	Variation (%)
(a) COI				
AMOVA without groups				
Among population without groups	13	146.743	0.48	46.01**
Within populations	305	171.69	0.563	53.99
Total	318	318.433	1.043	
AMOVA pooled by local basin				
Among groups	6	101.792	0.117	11.07
Among pops within groups	7	45.689	0.375	35.40**
Within pops	305	172.58	0.566	53.53**
Total	318	320.061	1.057	
AMOVA pooled by ocean				
Among groups	2	72.408	0.348	28.91*
Among populations within groups	11	74.335	0.293	24.36**
Within populations	305	171.433	0.563	46.74**
Total	318	318.433	1.204	
(b) ANT				
AMOVA without groups				
Among population without groups	13	105.087	0.146	8.25**
Within populations	616	1001.645	1.626	91.75
Total	629	1106.733	1.772	
AMOVA pooled by local basin				
Among groups	6	48.09	**−**0.005	**−**0.36
Among pops within groups	7	35.152	0.12	8.55**
Within populations	616	796.634	1.293	91.81**
Total	629	879.876	1.409	
AMOVA pooled by ocean				
Among groups	2	39.031	0.072	4.00
Among pops within groups	11	66.057	0.106	5.88**
Within populations	616	1001.645	1.626	90.11**
Total	629	1106.733	1.804	

The Mantel test detected a significant, though slight, correlation between genetic distance and the log of geographic distance when including all samples (*r *=* *0.125, *P* < 0.0001). When considering only Atlantic populations (the most heavily sampled region), the Mantel test also detected a correlation between genetic and geographic distance (*r *=* *0.123, *P* <0.0001). Divergence between populations could result in false positives in this test (Meirmans 2012). Private alleles from each ocean basin did display the highest divergence detected in our study (COI‐H in the Pacific and COI‐E in the Gulf of Mexico show a minimum of seven nucleotide changes, Fig. 2) and could affect our isolation by distance results.

A total of 37 (51%) significant population comparisons were detected at both the *F*_ST_ and Φ_ST_ statistics (Tables 4 and 5). Of these 37, 60% were between oceans and 40% were within the Atlantic (the only basin containing significant comparisons between populations). Several population comparisons between distant regions were not significant. Φ_ST_ values among populations ranged from −0.045 to 0.937. In the Atlantic, Φ_ST_ ranged from −0.045 to 0.877, while the Pacific populations ranged from −0.026 to 0.208.

**Table 4:** *F*_ST_ values between populations of *Botrylloides nigrum* at the COI (lower diagonal) and ANT (upper diagonal) loci. Asterisks represent significant comparisons after Bonferonni correction (*P *<* *0.004)

	IR	TB	TX	VER	BZ	PR	VZ	BOC	BR	MAZ	PA	HI	IS
IR	–	0.012	0.091	0.201*	0.054	0.106*	0	0.016	0.012	0.01	0.019	0.005	0.073*
TB	−0.013	–	0.162*	0.270*	0.053	0.157*	0.023	0.063	0.052	0.005	0.011	0.036	0.109*
TX	0.003	−0.06	–	0.02	0.275*	−0.006	0.137	0.008	0.022	0.171*	0.198*	0.06	0.090*
VER	0.500*	0.381*	0.321*	–	0.409*	0.052	0.274*	0.096*	0.093*	0.271*	0.296*	0.194*	0.095*
BZ	0.021	0.087	0.133	0.636*	–	0.266*	−0.01	0.133*	0.136*	0.078	0.073	0.064	0.230*
PR	0.267*	0.144	0.086	0.072	0.359*	–	0.171*	0.048	0.034	0.147*	0.180*	0.078*	0.113*
VZ	−0.043	−0.035	−0.025	0.490*	−0.025	0.196	–	0.031	0.057	0.061	0.06	−0.001	0.138
BOC	0.658*	0.605*	0.595*	0.053	0.839*	0.231*	0.774*	–	−0.003	0.079	0.093*	0.009	0.052
BR	0.004	0.067	0.115	0.631*	−0.028	0.379*	−0.016	0.789*	–	0.041	0.057*	0.029	0.03
MAZ	0.666*	0.610*	0.604*	0.08	0.862*	0.233*	0.797*	−0.023	0.803*	–	−0.022	0.048	0.098*
PA	0.708*	0.682*	0.695*	0.134	0.900*	0.297*	0.864*	0.001	0.836*	0	–	0.065*	0.103*
HI	0.647*	0.586*	0.566*	0.036	0.811*	0.218*	0.737*	−0.028	0.770*	−0.014	0.011	–	0.118*
IS	0.399*	0.273*	0.208	0.006	0.541*	0.035	0.395	0.127	0.532*	0.156	0.208	0.114	–

**Table 5:** Φ_ST_ values between populations of *Botrylloides nigrum* at the COI (lower diagonal) and ANT (upper diagonal) loci. Asterisks represent significant comparisons after Bonferonni correction (*P *<* *0.004)

	IR	TB	TX	VER	BZ	PR	VZ	BOC	BR	MAZ	PA	HI	IS
IR	–	0.012	0.091	0.201*	0.054	0.106*	0	0.016	0.012	0.01	0.019	0.005	0.073*
TB	−0.013	–	0.162*	0.270*	0.053	0.157*	0.023	0.063	0.052	0.005	0.011	0.036	0.109*
TX	0.003	−0.06	–	0.02	0.275*	−0.006	0.137	0.008	0.022	0.171*	0.198*	0.06	0.090*
VER	0.500*	0.381*	0.321*	–	0.409*	0.052	0.274*	0.096*	0.093*	0.271*	0.296*	0.194*	0.095*
BZ	0.021	0.087	0.133	0.636*	–	0.266*	−0.01	0.133*	0.136*	0.078	0.073	0.064	0.230*
PR	0.267*	0.144	0.086	0.072	0.359*	–	0.171*	0.048	0.034	0.147*	0.180*	0.078*	0.113*
VZ	−0.043	−0.035	−0.025	0.490*	−0.025	0.196	–	0.031	0.057	0.061	0.06	−0.001	0.138
BOC	0.658*	0.605*	0.595*	0.053	0.839*	0.231*	0.774*	–	−0.003	0.079	0.093*	0.009	0.052
BR	0.004	0.067	0.115	0.631*	−0.028	0.379*	−0.016	0.789*	–	0.041	0.057*	0.029	0.03
MAZ	0.666*	0.610*	0.604*	0.08	0.862*	0.233*	0.797*	−0.023	0.803*	–	−0.022	0.048	0.098*
PA	0.708*	0.682*	0.695*	0.134	0.900*	0.297*	0.864*	0.001	0.836*	0	–	0.065*	0.103*
HI	0.647*	0.586*	0.566*	0.036	0.811*	0.218*	0.737*	−0.028	0.770*	−0.014	0.011	–	0.118*
IS	0.399*	0.273*	0.208	0.006	0.541*	0.035	0.395	0.127	0.532*	0.156	0.208	0.114	–

When populations were pooled by ocean basin, the Pacific Ocean showed significantly negative values at Tajima's D, Fu and Li's F, and Fu and Li's D (Table 6). Populations in Bocas, Panama (Atlantic) and Oahu, Hawaii (Pacific) also revealed statistically negative values. Texas (Atlantic) displayed a significantly negative value for Tajima's D. Negative values suggest that either selection or demographic factors may be reducing genetic diversity of COI in these populations.

**Table 6:** Demographic and selection parameters for *Botrylloides nigrum* populations at each locus, estimated by population and by ocean

Site	COI					ANT				
	*D*	*Fs*	F*	D*	*R*_2_	*D*	*Fs*	F*	D*	*R*_2_
ATL	0.777	2.315	0.0288	−0.399	0.113	1.091	2.04	1.499	1.333	0.125
IR	1.467	5.424	1.013	1.013	0.186	0.216	2.225	1.158	1.379	0.107
TB	1.935	5.141	1.539	1.102	0.233	−0.391	1.882	0.018	0.21	0.111
TX	2.008*	4.59	1.581	1.178	0.256	0.337	3.203	−0.589	−0.865	0.154
VR	1.249	2.719	1.058	1.249	0.144	2.145*	5.772	1.882*	1.364	0.209
BZ	−0.908	1.187	0.127	0.481	0.111*	0.216	0.665	0.564	0.594	0.144
PR	1.843	2.573	1.572	1.151	0.214	0.896	2.646	0.972	0.791	0.163
VZ	−1.486	0.668	−1.683	−1.567	0.277	0.842	0.944	0.846	0.716	0.22
BOC	−1.887*	0.707	−2.996*	−2.868*	0.192	0.039	1.624	0.63	0.778	0.124
BR	0.094	3.554	0.884	1.041	0.126	1.525	4.58	1.655	1.339	0.164
PAC	−1.949*	−1.905	−3.962**	−4.046**	0.095	0.109	2.331	0.103	0.126	0.111
MAZ	−	−	−	−	−	0.493	3.547	0.814	0.786	0.144
PA	−	−	−	−	−	0.447	3.355	0.769	0.754	0.137
HI	−2.008*	−0.747	−3.368**	−3.269**	0.145	0.145	1.506	0.381	0.411	0.13
MED/IS	1.411	5.054	1.344	1.051	0.194	1.893	5.754	1.845*	1.39	0.178
Total	0.6	1.94	−0.616	−1.164	0.1	0.916	1.11	0.912	0.646	0.119

Neutrality statistics: Tajima's D (*D*), Fu's Fs (*Fs*), Fu and Li's F (F*), Fu and Li's D (D*), and Ramos‐Onsins & Rozas's statistic (*R*_2_). Asterisks represent significance (*P <* *0.05, **P <* *0.02).

Coalescent estimates of gene flow (effective number of migrants per generation) between ocean basins were high (4.4–433.7). Gene flow was lower from the Atlantic and Mediterranean to the Pacific (4.4 and 5.1, respectively), while all other directions showed very high gene flow (270.6–433.7, Table 7). Very high estimates of gene flow may reflect limited resolution due to low sampling of populations and low diversity at the COI locus. For instance, migration estimates from the Pacific and Mediterranean to other basins may be biased upwards because these locations contain mainly shared common alleles, and the Pacific population displays a loss of rare alleles. The highest theta values were detected in the Atlantic (0.0515) and Mediterranean (0.0517) populations, while the lowest was in the Pacific (0.0003).

**Table 7:** MIGRATE estimates of theta (*θ*), the mean and standard deviation of migration (*m*) after five analyses, and the effective number of migrants (ANT 0.25*θm,* COI *θm*) for each ocean basin (A = Atlantic, P = Pacific, M = Mediterranean)

	A (*θ*)	P (*θ*)	M (*θ*)	P > A	M > A	A > P	M > P	A > M	P > M
COI									
Mean (m)	0.0515	0.0003	0.0517	5249.8	6118.7	1763.9	2007.7	5600.7	8384.7
SD (m)	0.0042	0.0035	0.001	1193.4	181.5	186.6	165.4	400.5	105.8
Nm				270.6	315.4	4.4	5.1	289.7	433.7
ANT									
Mean (m)	0.0292	0.0459	0.0022	5615.1	6566.7	6265.4	6808.8	3893.8	3620.2
SD (m)	0.0162	0.006	0.0007	1034.4	1167.3	445.9	513.5	286.1	269.1
Nm				164.1	191.9	287.8	312.7	8.7	8

### Heteroplasmy in COI

Six individuals of 321 total showed double peaks in COI sequences at nucleotide (nt) positions 100, 325, 336, and 410, which were consistent across three repeated extractions (Table S2). We interpret heteroplasmy detected in this study as chimerism for the following reasons: (1) extra alleles were detected in mtDNA and thus would not be an artifact of extracting DNA from fertilized eggs or larval tissue (2) some heteroplasmic individuals contained only common COI alleles, (3) fusion is common in some natural populations (Westerman et al. 2009), and (4) chimeras have been previously reported in a COI study of the social ascidian *Perophora japonica* (Pérez‐Portela et al. 2012). Therefore, chimeric individuals were included in our analyses and were counted as two individuals. We detected a 1.87% rate of field chimerism in this study, which should be considered a very conservative estimate due to the low diversity seen at the COI locus.

The polymorphism at position 100 was found in other nonchimeric individuals in the dataset. The other three polymorphisms (at nt positions 325, 336, 410) represented new diversity only detected in chimeras. The substitution at position 325 was a synonymous mutation. Position 336 showed a nonsynonymous mutation that was found in one individual (HI19) and changed a leucine to phenylalanine (score of −3 Blosum 62 Amino Acid Similarity Matrix). Two individuals from the same location (PR11 & PR 24) also contained a nonsynonymous mutation at position 411 that changed arginine to serine (score of 1 Blosum 62 Amino Acid Similarity Matrix). This diversity represented three COI haplotypes (COI‐D, E, H, Table S2). No more than two alleles were detected in any chimeric individuals at the ANT locus.

Chimeric individuals were discovered in several locations across two oceans. Three chimeric individuals were detected in Puerto Rico, a site with four haplotypes detected in 25 total individuals. Veracruz, Mexico (three haplotypes, *n *=* *18) contained two chimeric individuals, and Hawaii (three haplotypes, *n *=* *30) had one chimeric individual. When chimeric individuals were removed from population diversity analyses, Puerto Rico still remained one of the most diverse populations (Hd: 0.481 ± 0.009, *π*: 0.003 ± 0.002). Conversely, after removing chimeras, diversity greatly decreased in Hawaii (Hd: 0.069 ± 0.004, *π*: 001 ± 0.000) and Veracruz (Hd: 0.000, *π*: 0.000), which can affect interpretation of genetic diversity of these populations compared to other locations (see Discussion).

### Nuclear adenine nucleotide translocase (ANT) gene

We recovered a 265 nt fragment of the inferred ANT gene from 319 individuals from 16 locations. The ANT locus has an intron in some species, although not in the tunicates *Halocynthia roretzi* (Jarman et al. 2002) or *Styela plicata* (Pineda et al. 2011). Here, we were able to translate all amino acids of our amplified fragment and align to *Halocynthia roretzi* (Genbank Accession D83069) and a smaller fragment from *Styela plicata* (Genbank Accession HQ916363). The ANT exon dataset contained 13 polymorphic sites (5%) that were all synonymous substitutions. No recombination events were detected within ANT sequences.

The *F*_IS_ coefficient revealed significant inbreeding at the ANT locus when considering all populations (0.148, Table 2). When *F*_IS_ was calculated for each population, Tampa Bay (0.525), Veracruz (0.271), Venezuela (1.000), Bocas (0.184), and Israel (0.167) each showed significant inbreeding. The Atlantic was the only ocean basin with a significant *F*_IS_ (0.193) value. Texas (−0.189) and Hawaii (−0.251) showed significantly negative *F*_IS_ values.

**Figure 3:**
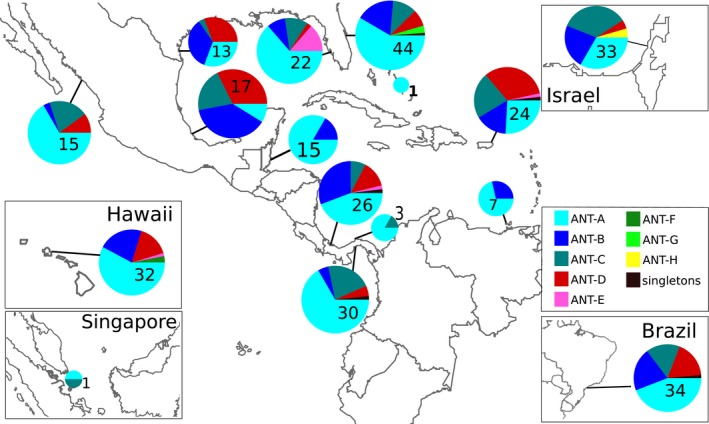
Map showing sampling sites of *Botrylloides nigrum* and ANT alleles. Circles represent populations, the size of circles and the numbers within circles represents sample size, and colors represent alleles. Singletons are shown in black.

We chose an inferred probability for phase acceptance of < 97% for all samples, which resulted in discarding two low‐resolution samples from the study (317 total samples were used in analyses). In total, 13 alleles were discovered and four of these were found in each sampled ocean basin (Table 2, Fig. 3). The Atlantic contained ten total including three private alleles, the Pacific had seven total alleles including one private, and the Mediterranean contained five alleles with one being private. Total gene diversity was 0.674 (±0.014 SD) and total nucleotide diversity was 0.0099 (±0.0059 SD). Basins within the Atlantic contained populations with the highest diversity measures, similar to COI results. In general, Pacific populations had slightly lower allelic richness, haplotype diversity, and nucleotide diversity compared to populations in the Atlantic. After rarefaction (to *n *=* *66), allelic richness values were similar: the Atlantic Ocean had the highest allelic richness (4.194), followed by the Pacific (4.098) and the Mediterranean (4.000).

**Figure 4:**
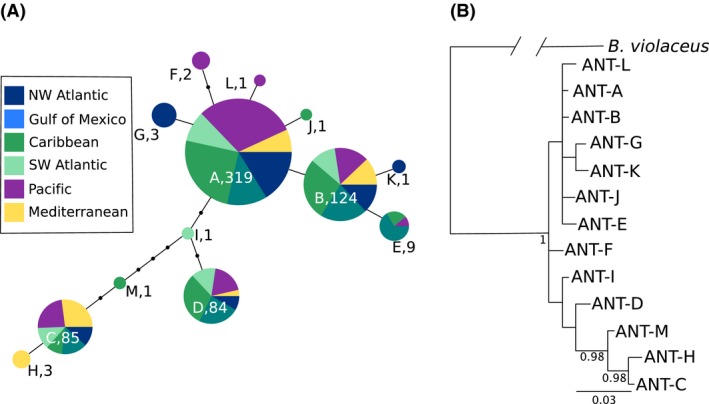
(A) Minimum spanning network for the ANT locus. Circles are labeled with the haplotype name and number of individuals with that haplotype, size differences of circles reflect frequency, and colors represent regions where the haplotype was found. (B) Phylogeny of the 265‐bp region of ANT locus using Bayesian analysis. Nodes with posterior probabilities >0.95 are shown.

Similar to COI, an AMOVA revealed the greatest source of genetic variation to be within populations (91.75%, Table 3). Unlike COI, population structure was not detected among ocean basins; each ocean shared several alleles that were abundant and detected at similar frequencies (Fig. 3). The Mantel test revealed a signific‐ant yet slight correlation between genetic divergence and geographic distance when comparing all populations (*r* = 0.095, *P *=* *0.001). Additionally, we did not detect high divergence among basins that could bias these results (Fig. 4).

The mean divergence among ANT alleles was 1.0%. Similar to COI, Bayesian analysis could not resolve the relationships of ANT alleles with high support. Pairwise *F*_ST_ and Φ_ST_ comparisons revealed fewer (22) significant comparisons between populations at the ANT locus than was found among COI comparisons (40, Tables 4 and 5). The majority of significant comparisons (50%) were found between populations within the Atlantic, while 45% were between Atlantic and Pacific populations. Only one significant population comparison was detected within the Pacific region. Overall Φ_ST_ values ranged from −0.022 to 0.299, lower than Φ_ST_ values seen between populations at the COI locus. In the Atlantic they ranged from −0.004 to 0.299.

Unlike the COI allele, neutrality tests at ANT did not reveal an effective population decrease across Pacific populations (Table 4). Veracruz showed statistically significant positive values at Tajima's D and Fu and Li's F statistics. Israel also showed a significant positive value at Fu and Li's F that may represent population growth or diversifying selection in these areas.

Estimates of gene flow calculated with the ANT locus were also high (2.0–78.2). The lowest gene flow was detected from the Atlantic and Pacific to the Mediterranean (2.2 and 2.0, respectively, Table 7), which may be due to reduced sampling in this location. Higher allelic diversity and neutral demographic patterns in populations at the ANT locus may offer more accurate estimates of gene flow compared to the COI locus. Theta estimates for the Pacific (0.046) and Atlantic (0.029) were an order of magnitude higher than the Mediterranean (0.002), though this could be due to uneven sampling.

## Discussion

### A single species detected globally

Despite the broad and environmentally variable distribution of *Botrylloides nigrum*, there was no evidence of cryptic species diversity. Cryptic lineages have been detected in many, though not all, ascidian species displaying broad environmental ranges (Stefaniak et al. 2009; Bock et al. 2010, 2012; Zhan et al. 2010; Pineda et al. 2011; Teske et al. 2011; Pérez‐Portela et al. 2013). Instead, we found a single species with low overall COI haplotype divergence (0.4%) that has established in the Atlantic, Mediterranean, Pacific, and Indo‐Pacific basins. This study also confirmed *B. nigrum* in three previously suspected locations (based on morphological comparisons): marinas in Oahu, Hawaii; marinas on the Pacific coast of Mexico from Mazatlan to Nayarit; and on buoys in shipping lanes off the coast of Singapore. Also, all samples from the Mediterranean coast of Israel genotyped as *B. nigrum* and not as *Botrylloides leachii,* as recently proposed (Brunetti 2009).

### Global phylogeography

Global COI gene (Gd = 0.518) and nucleotide (*π *= 0.0038) diversity of *B. nigrum* are low compared to *Botryllus schlosseri* (partially global sampling, Gd = 0.872 and *π *= 0.054; introduced range Gd = 0.874, *π *= 0.012) but similar to *Botrylloides violaceus* (partially global Gd = 0.461 and *π *= 0.007; introduced range Gd = 0.384, *π *= 0.006) (Lejeusne et al. 2011). The high overall diversity of *Botryllus schlosseri* populations was considered a reflection of long‐term global establishment, multiple introductions from various sources, and a large native geographic region (Yund et al. 2015); while the lower diversity detected in *Botrylloides violaceus* populations was suggested to result from a recent widespread invasion from a single source as well as possible undersampling of the native range. Additionally, global ANT gene (0.674) and nucleotide (0.0099) diversity of *B. nigrum* are much lower than a confamilial, *Styela plicata* (global Gd = 0.820 and *π *= 0.03214), which is thought to have established hundreds of year ago and now displays a global distribution with repeated genetic shuffling (Carlton 2009; Pineda et al. 2011). Another phylogeographic study of the introduced tunicate, *Pyura stolonifera*, found 20 COI alleles and 30 ANT alleles (compared to 8 COI and 13 ANT here) across the southern hemisphere (Teske et al. 2011). Despite low global diversity, *B. nigrum* populations revealed a slight pattern of isolation by distance at both loci, a similar pattern to *Botryllus schlosseri*, but not to *Botrylloides violaceus* global populations.

Tunicates are a fast‐evolving group with high genetic variation (Tsagkogeorga et al. 2012) and we originally expected to find higher diversity across sampled populations. However, the low allelic and nucleotide diversity detected at both COI and ANT in *B. nigrum* shows a single species that is possibly bottlenecked across its distribution. However, alleles that are regionally restricted were found at both loci. This could be a signal of an intermediate introduction stage between the very recent spread of *Botrylloides violaceus* and the older establishment of *Botryllus schlosseri* and *Styela plicata* populations (Lejeusne et al. 2011; Pineda et al. 2011). Additionally, similar low diversity detected in *Botrylloides violaceus* populations may indicate a more recently diverged genus with low diversity (Saito et al. 2001) or a lower rate of evolution for this genus. Estimates of mitogenomic variability of the *Botrylloides* genus are lower than the *Botryllus* and *Styela* genera (Griggio et al. 2014).

In addition to timescale differences in introduction events of these closely related species, introductions into highly diverse tropical marine regions may be more difficult and happen less frequently compared to temperate regions. Biodiversity has been suggested as a barrier to invasions where crowding, space competition, and strong predation pressures can limit the establishment and spread of introduced species (Kennedy et al. 2002; Stachowicz et al. 2002; Freestone et al. 2013). However, *B. nigrum* displays a widespread distribution across global tropical regions and does not seem to be more restricted than temperate congeners. The low global genetic diversity and divergence detected was similar to one temperate species in this clade, and thus, may not reflect selective differences that might be expected in complex tropical environments. More studies of tropical species are needed to understand if and what differences there may be in the genetic patterns of introductions into tropical versus temperate regions.

### Signal of past demographic events or selection

Even though we detected low overall diversity at both loci sampled in this study, we only found significant negative neutrality statistics at the COI locus. This may signal a very recent introduction to the Atlantic coast of Panama, the Pacific coast of Panama and Mexico, and in Hawaii. Molecular studies often find low genetic diversity in introduced populations because only a few individuals from the source may successfully arrive or establish because of either local selection or stochastic allele loss (Sakai et al. 2001). Additionally, small populations (such as new introductions) with limited dispersal are sensitive to allele surfing, where alleles of individuals at the frontier of an expansion increase in frequency (Excoffier et al. 2009). The COI‐A allele is common in all sampled regions in this study, and its very high frequency in the Pacific could be a result of an introduction of only a few individuals from another region. While we did not detect deviation from neutrality or regional structure at the ANT locus in these locations, a bottleneck may have differentially affected the mitochondrial and nuclear loci. Mitochondrial genes have smaller effective population sizes that can make them more sensitive to demographic effects (Shaw et al. 2004).

Alternatively, reduced COI diversity observed in some populations could be selection on the COI locus or a linked gene. While mitochondrial genes have been widely employed as neutral markers in phylogeographic studies, mitochondrial genes are not subject to recombination and are particularly susceptible to reflecting the influence of selection pressures or genetic hitchhiking events (Ballard and Kreitman 1995). Additionally, these genes play an important role in metabolism, and variation within them may affect physiological differences between individuals (Ballard and Pichaud 2014). In a highly invasive ascidian, *Didemnum vexillum*, two distinct mitochondrial clades have been detected: one is an aggressive global invader, and one is restricted to the native range of Japan. Two potentially functional changes in the mitochondrial genome were found between the globally invasive clade and the Japan‐restricted clade, which may affect the physiological differences and invasive potentials of each clade (Smith et al. 2015). Here, we did not detect nonsynonymous changes between COI‐A and COI‐B, suggesting their distribution and frequency differences among ocean basins are a result of stochastic sorting of limited introductions.

### Introduction pathways

By examining global shipping patterns, we can identify areas where exchange of species will likely accelerate in the future (Keller et al. 2011; Seebens et al. 2013). A major shipping pathway in the tropics is through the Panama Canal, where expansions are projected to increase introductions (Muirhead et al. 2015). Surveys have reported high numbers of introduced or cryptogenic species on each side of the Panama Canal, including *B. nigrum* (Carman et al. 2011; Schlöder et al. 2013). It is unclear whether the freshwater lake within the canal acts as a barrier to many organisms, including ascidians. Organisms fouling ship hulls are exposed to freshwater for at least 6–8 h when traveling through the canal (Cohen 2006). Sea chests (a recess in the hull of the ship) may buffer fouling individuals from some external environmental stressors (Cohen 2006; Coutts and Dodgshun 2007). Botryllid ascidians may be able to survive low salinity exposure during transit because of their incredible regeneration abilities—they are capable of regenerating whole zooid bodies from only small fragments of vascular tissue (Rinkevich et al. 1995), even in highly variable estuarine environments (Chow et al. 2012).

The Atlantic basin contained the highest diversity estimates and may be a source for introductions to other areas. Coalescent‐based migration estimates detected gene flow between all sampled basins, but fewer migrants to the Pacific at the COI locus (Table 7). Short‐term exposure to extreme salinity changes, like those encountered by fouling organisms on ships transiting the Panama Canal, could bottleneck populations through massive die‐offs or select for the most tolerant genotypes.

Other modes of transit could also facilitate exchange of individuals between these oceans. Organisms could also be translocated along with shellfish aquaculture, which provides habitat for invasive ascidians (Cohen et al. 2011), including *B. nigrum* (Rocha 2009). Local boat traffic and movement of docks can be a vector for small‐scale population expansion (Davidson et al. 2010; Clarke Murray et al. 2011; Darling et al. 2012) and could move specimens from populations established in farms to other areas. While aquaculture farms and local boats are geographically restricted compared to large‐scale shipping, the genetic connectivity of widespread *B. nigrum* populations displays a scale of introduction that reflects movement via shipping pathways combined with several local vectors.

### Native source

Introduced populations have historically been characterized by low genetic diversity due to bottlenecks associated with few individuals successfully establishing in new areas. However, many studies have shown that introduced populations can accumulate more diversity than native populations as a result of multiple introductions from various locations from the native range (Roman and Darling 2007). Additionally, undersampling the native range can lead to incorrect conclusions (Geller et al. 2010). These issues can complicate the ability to resolve the native source for many introductions. In related temperate species, high genetic diversity has been used to characterize the native ranges of both *Botryllus schlosseri* and *Botrylloides violaceus* (Lopez‐Legentil et al. 2006; Lejeusne et al. 2011). Conversely, genetic admixture has hindered the ability to detect the native source of the very old introduction of *Styela plicata* (Pineda et al. 2011).

For *Botrylloides nigrum*, an AMOVA shows that the global gene pool is admixed among the Pacific, Atlantic, and Mediterranean basins where the greatest source of diversity is found within populations. This admixture may hinder our ability to determine a native source. In the hypothesized native range, the West Atlantic, seven COI haplotypes were detected in 215 individuals. Fewer COI haplotypes were detected in the Pacific (three haplotypes in 75 individuals) and Mediterranean (two haplotypes in 31 individuals). Furthermore, the coalescent‐based program MIGRATE estimated high theta values –a more robust measure of genetic diversity— for the Atlantic population at both loci (COI = 0.052, ANT = 0.029). In comparison, theta values an order of magnitude lower were detected in the Pacific (COI locus = 0.0003) and Mediterranean (ANT locus = 0.0022) populations, a possible signal that these are introduced ranges. However, the West Atlantic is the most heavily sampled region in this study and the high diversity reported here could be biased by the over detection of rare alleles.

We hypothesize the Caribbean as the native range of *B. nigrum* because: (1) This is the only region displaying a continuous distribution, (2) At both markers, we consistently detected high genetic diversity in Puerto Rico and nearby populations, and (3) Pacific and Mediterranean regions are likely not the native source because of very low population diversity and reports are few and geographically scattered. However, the distribution of this species is likely greater than what is currently reported, and broader sampling in the Eastern Atlantic, Red Sea, and Indo‐ and West‐Pacific with higher resolution loci may help resolve the native range.

### Chimerism at COI

Rare alleles were discovered in what we determined to be chimeric individuals from Hawaii, Puerto Rico, and Veracruz, Mexico that were not detected in nonchimeric colonies. Though low detection of these genotypes could be a result of limited sampling, chimerism may act to increase genetic diversity of populations by hiding the genotype of one individual within the tissues of the other after fusion of distinct, but closely related, individuals. Botryllid chimeras are capable of retaining the genotypes of each fused colony and shifting which genotype takes over somatic growth when exposed to varying environments, perhaps allowing the colony to express the best‐fit genotype for the environment (Rinkevich and Yankelevich 2004). However, the gametic constituent appears unlinked to genotype shifts in the somatic tissue and the less environmentally tolerant genotype could be harbored and invisible to natural selection for a generation (Rinkevich 2005). The rare diversity detected in chimeras in this study could be a result of a genotype escaping environmental stress by taking refuge in the gametes or soma of a more tolerant colony. This ability could help populations retain higher genetic diversity, an important feature for the establishment and spread of introduced species.

Chimeras are commonly identified using highly polymorphic loci, including microsatellites (Ben‐Shlomo et al. 2001; Paz et al. 2003). They have also previously been identified at the COI locus in the ascidian *Perophora japonica* (Pérez‐Portela et al. 2012), but detection with COI is rare. We speculate that a lack of reports using COI may be because of its low diversity or detection issues during sequencing. We found that double peaks were occasionally difficult to distinguish from sequencing noise, and confirmation was only possible after repeating DNA extractions, amplifications, and sequencing. Therefore, ambiguous sites in the sequence reads of chimera‐forming species should be carefully considered. To interpret diversity of chimeras, we found cloning to be an effective detection method as chimerism rates in COI were low in our study, chimeras contained rare diversity, and heterozygous sites were occasionally ambiguous in sequence reads. Future studies using the COI gene in chimera‐forming species should be aware of the likelihood of chimeric individuals and the experimental biases that can disguise their detection.

Estimates of natural rates of fusion are essential to understanding the outcomes of fusion and why this life history strategy has evolved. This study offers the first estimate of genetically detected chimerism in wild populations (approximately 2% globally) of a tropical botryllid. The role of fusion and its rates may differ between native and introduced populations. Microsatellite studies of adult *Botryllus schlosseri* populations have estimated chimerism in the field at rates from 0.2 to 0.8% in the native range collected on natural substrates (Paz et al. 2003) and 3–29% in introduced populations in South America (Ben‐Shlomo et al. 2010) and New Zealand (Ben‐Shlomo et al. 2001). Our detection of fusion suggests that these populations have high densities or low diversity (as fusion only occurs between very closely related kin in this system).

### Impacts of a tropical invader

Molecular tools haven proven to be an invaluable resource for diverse management practices, and should be further employed to help unravel the extent of introduced species, particularly in the tropics (von der Heyden et al. 2014). Despite ecological and economic impacts of closely related temperate botryllids, the introduction of *B. nigrum* across global warm waters has been largely unexplored to date. The geographically widespread and environmentally variable distribution of this species suggests that it may tolerate a wide range of suitable environmental conditions (Table 1). Therefore, *B. nigrum* may be an especially useful model species to evaluate tropical invasions. It should also be considered a priority species when surveying for new or unresolved introductions in tropical and subtropical latitudes. Additionally, the distribution and genetic structure of this species is consistent with introductions through the Panama Canal. As expansion projects estimated to complete in 2016 are expected to greatly increase the shipping capacity of the Panama Canal (Reagan 2007; Muirhead et al. 2015), it is especially timely to establish baseline data for this and other introduced species around the canal, in order to evaluate (1) possible changes in diversity associated with the Canal expansion, and (2) the potential for secondary spread from this important global shipping hub.

## Conflict of Interest

None declared.

## Data Accessibility

Novel DNA sequences from this study are published in the NCBI Genbank database under the accession numbers KU711782 – KU711802.

## Supporting information


**Figures S1–S8.** Photographs of color morphotypes detected in this study.
**Table S1.** Occurrences of COI haplotypes and ANT alleles by locality.
**Figure S1.** White on pink color morphotype from Bocas del Toro, Panama. Photo by: E Sheets (San Francisco State University).
**Figure S2.** Yellow on red color morphotype from Indian River Lagoon, FL, USA. Photo by: L Walters (University of Central Florida).
**Figure S3.** Yellow on red color morphotype from Indian River Lagoon, FL, USA. Photo by: L Walters (University of Central Florida).
**Figure S4.** White on black color morphotype from Indian River Lagoon, FL, USA. Photo by: L Walters (University of Central Florida).
**Figure S5.** Bright orange on dark orange color morphotype from Oahu, Hawaii, USA. Photo by: C Craig (San Francisco State University).
**Figure S6.** Orange on black color morphotype from Oahu, Hawaii. Photo by: C Craig (San Francisco State University).
**Figure S7.** Yellow on black color morphotype from Veracruz, Mexico. Photo by: H Bahena (ECOSUR, Mexico).
**Figure S8.** White on red color morphotype from Veracruz, Mexico. Photo by: H Bahena (ECOSUR, Mexico).
**Table S2.** Chimeric individuals detected in this study.
