# Supplementary material for: Investigating the widespread introduction of a tropical marine fouling species
Source: Ecol Evol. 2016 Mar 11;6(8):2453–71. doi: 10.1002/ece3.2065 (PMC4788974; doi:10.1002/ece3.2065)
Supplement: Supplementary file 1 — Figures S1–S8. Photographs of color morphotypes detected in this study. Table S1. Occurrences of COI haplotypes and ANT alleles by locality. Figure S1. White on pink color morphotype from Bocas del Toro, Panama. Photo by: E Sheets (San Francisco State University). Figure S2. Yellow on red color morphotype from Indian River Lagoon, FL, USA. Photo by: L Walters (University of Central Florida). Figure S3. Yellow on red color morphotype from Indian River Lagoon, FL, USA. Photo by: L Walters (University of Central Florida). Figure S4. White on black color morphotype from Indian River Lagoon, FL, USA. Photo by: L Walters (University of Central Florida). Figure S5. Bright orange on dark orange color morphotype from Oahu, Hawaii, USA. Photo by: C Craig (San Francisco State University). Figure S6. Orange on black color morphotype from Oahu, Hawaii. Photo by: C Craig (San Francisco State University). Figure S7. Yellow on black color morphotype from Veracruz, Mexico. Photo by: H Bahena (ECOSUR, Mexico). Figure S8. White on red color morphotype from Veracruz, Mexico. Photo by: H Bahena (ECOSUR, Mexico). Table S2. Chimeric individuals detected in this study. [file ECE3-6-2453-s001.docx]

Supplementary Material

**Investigating the widespread introduction of a tropical marine fouling species**

Elizabeth A. Sheets, C. Sarah Cohen, Gregory M. Ruiz, Rosana M. da Rocha

Figures S1-8. Color morphotypes of *Botrylloides nigrum* collected from various locations in this study.


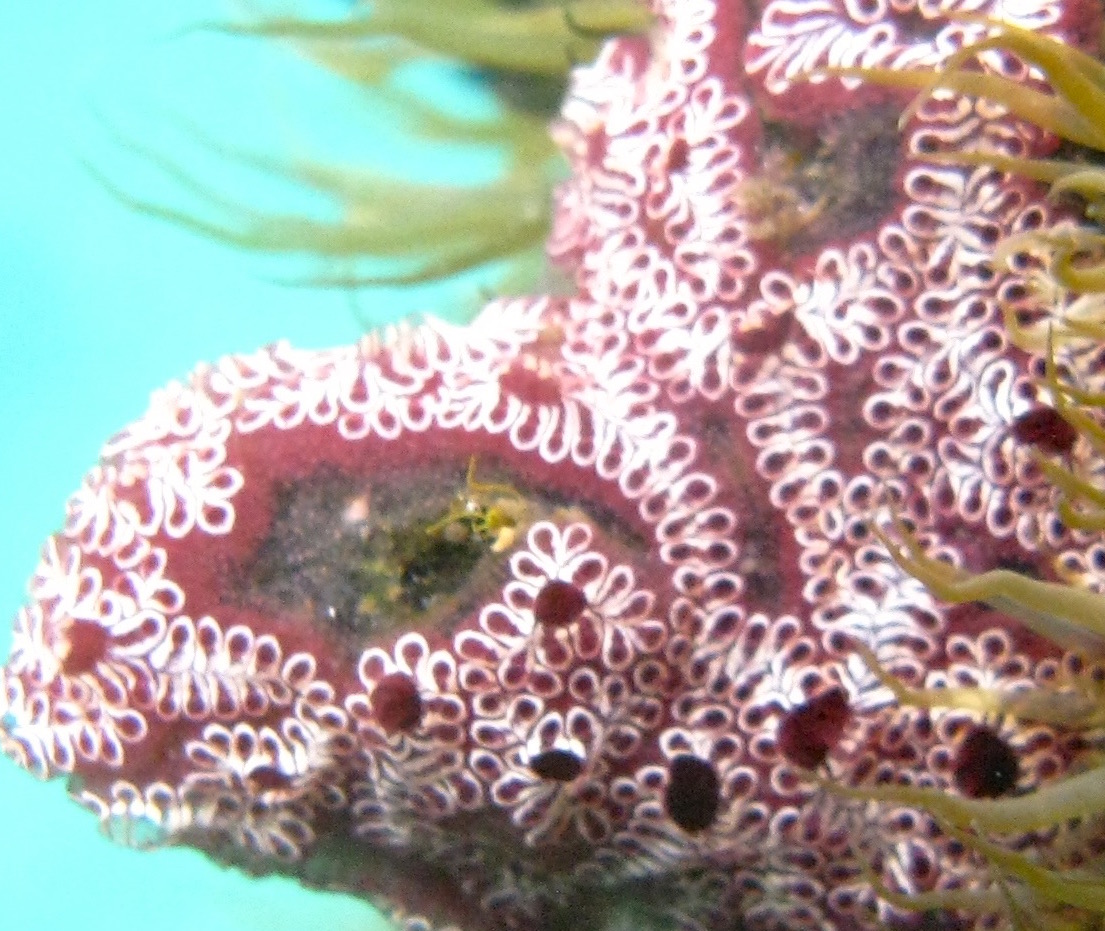


Figure S1. White on pink color morphotype from Bocas del Toro, Panama. Photo by: E Sheets (San Francisco State University)


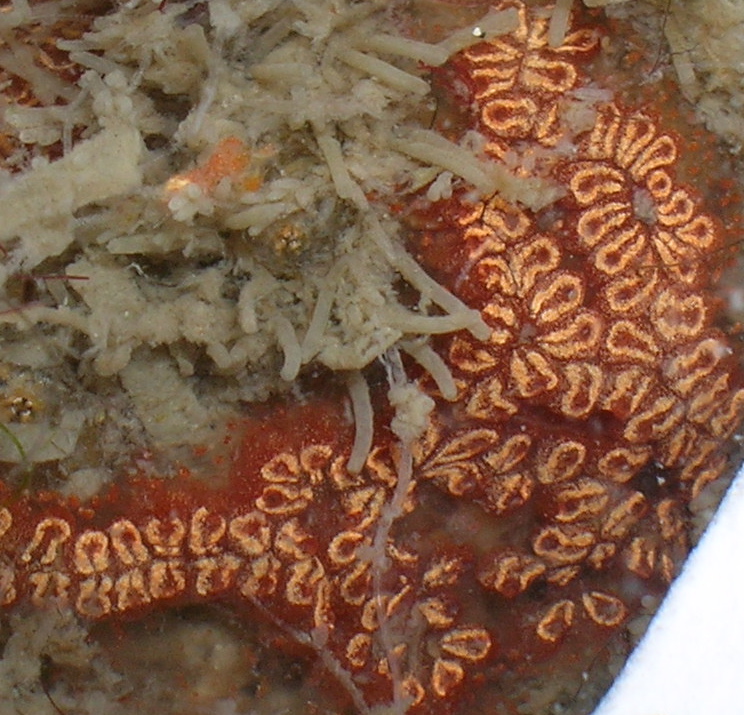


Figure S2. Yellow on red color morphotype from Indian River Lagoon, FL, USA. Photo by: L Walters (University of Central Florida)


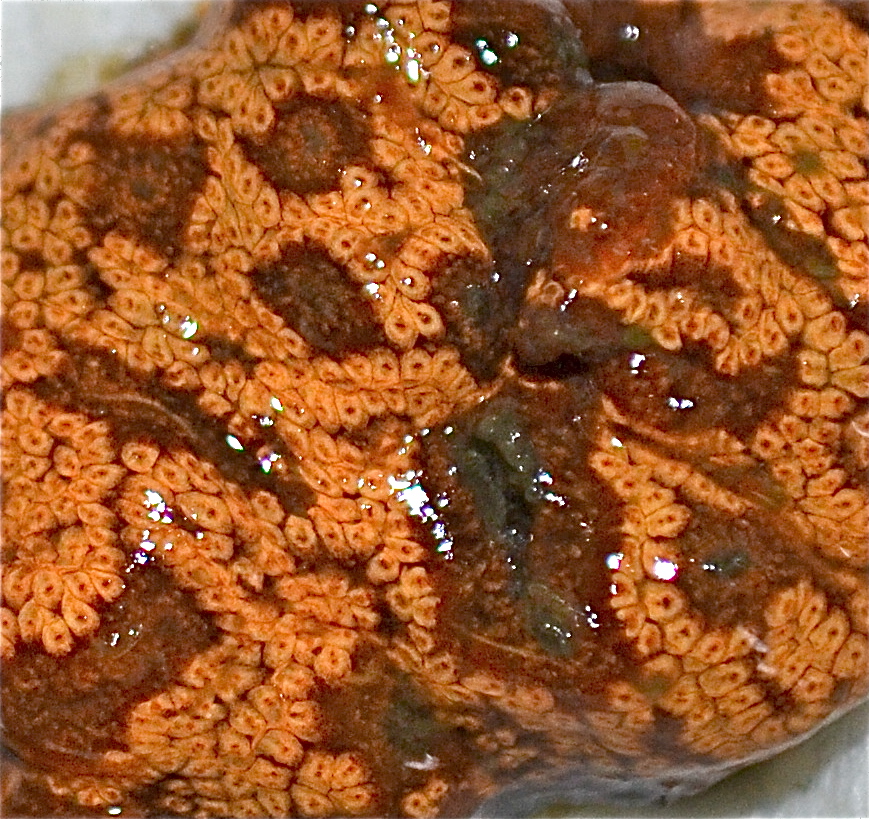


Figure S3. Yellow on red color morphotype from Indian River Lagoon, FL, USA. Photo by: L Walters (University of Central Florida)


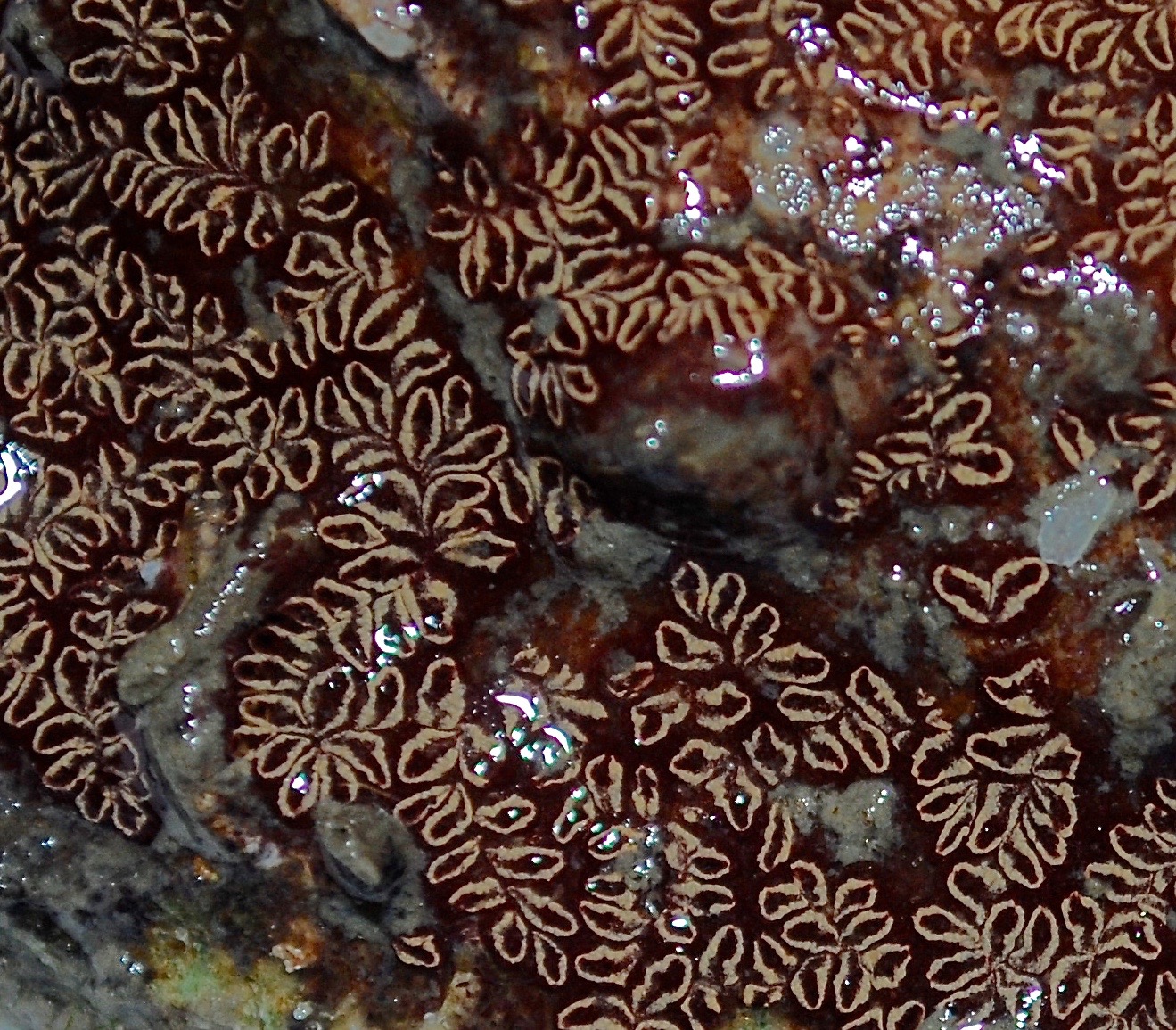


Figure S4. White on black color morphotype from Indian River Lagoon, FL, USA. Photo by: L Walters (University of Central Florida)


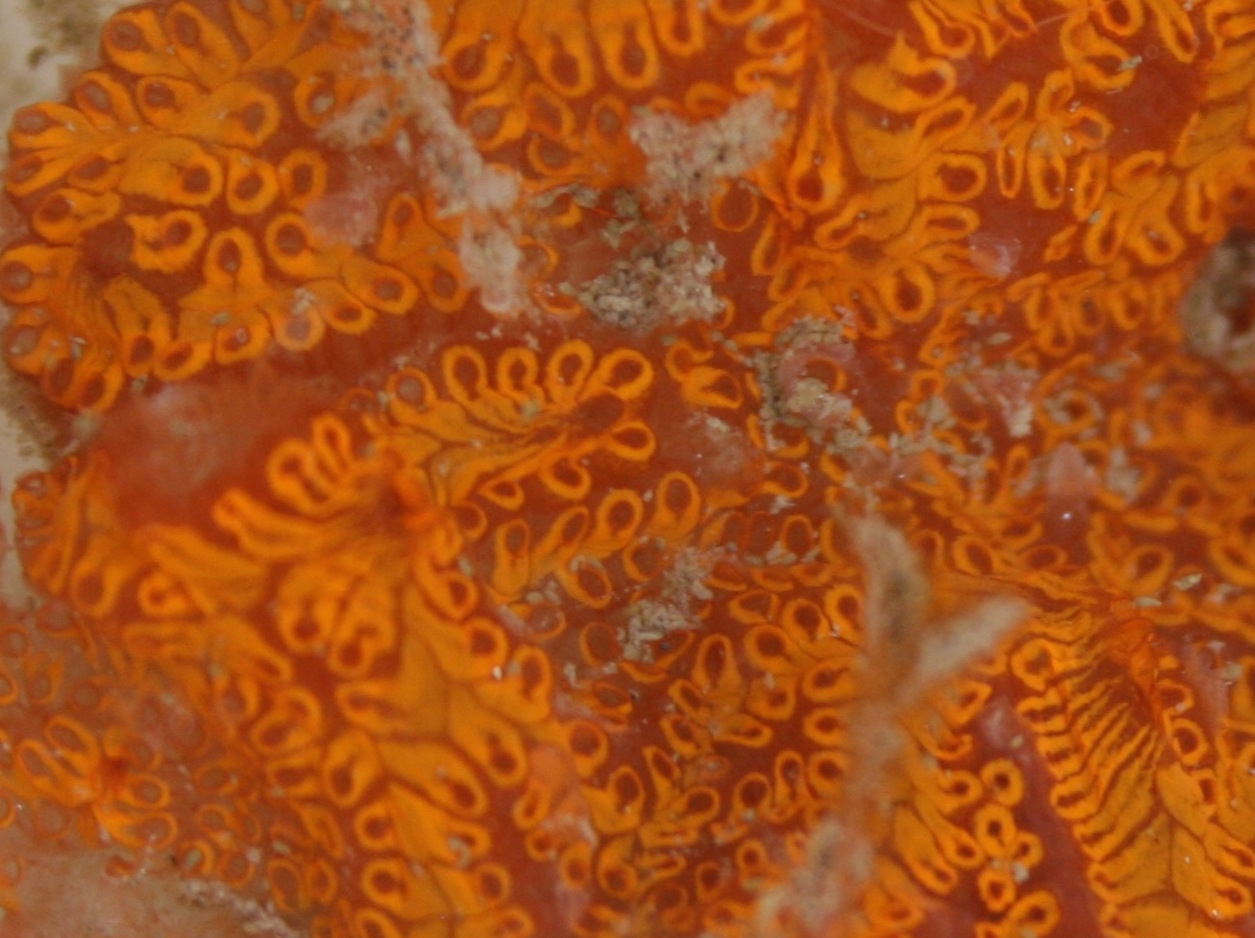


Figure S5. Bright orange on dark orange color morphotype from Oahu, Hawaii, USA. Photo by: C Craig (San Francisco State University)


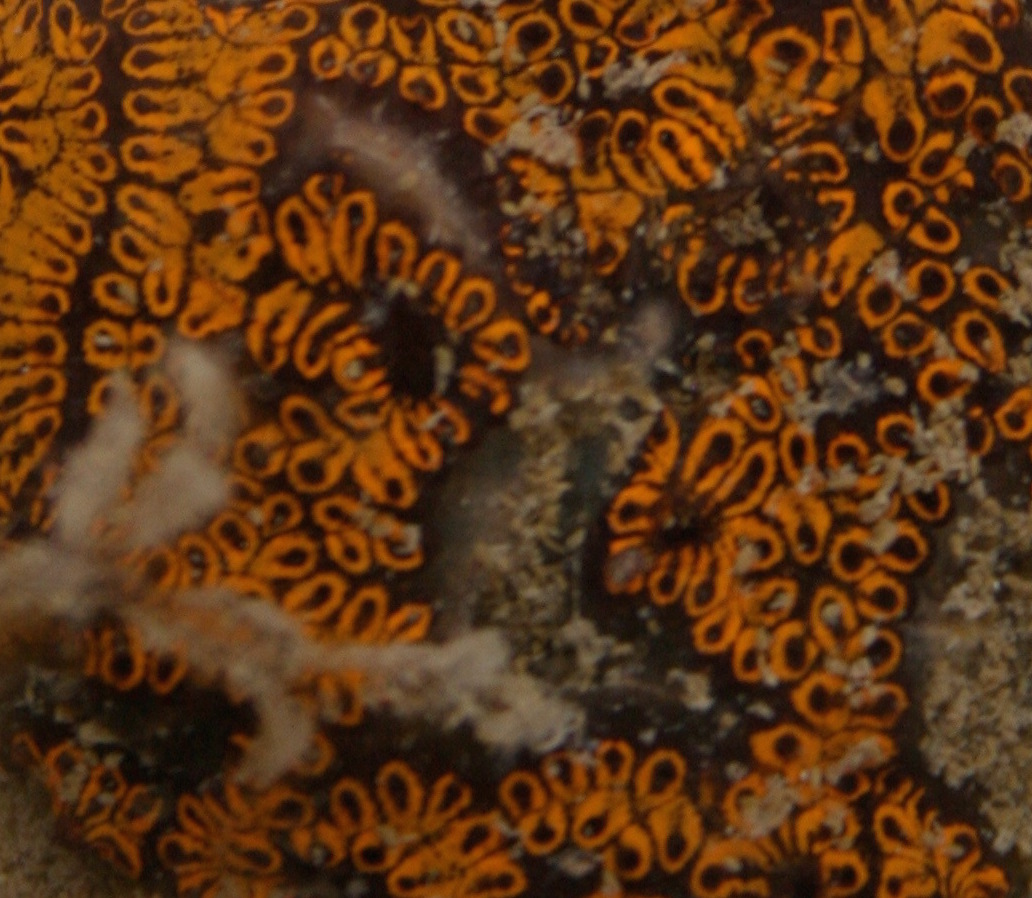


Figure S6. Orange on black color morphotype from Oahu, Hawaii. Photo by: C Craig (San Francisco State University)


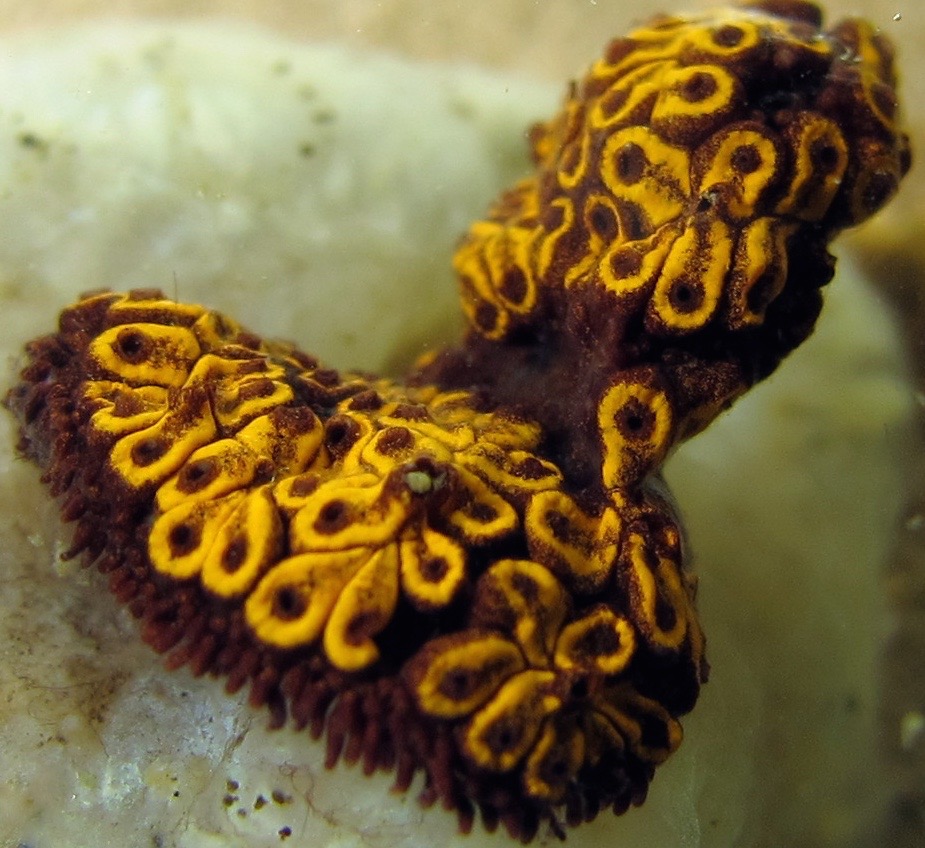


Figure S7. Yellow on black color morphotype from Veracruz, Mexico. Photo by: H Bahena (ECOSUR, Mexico)


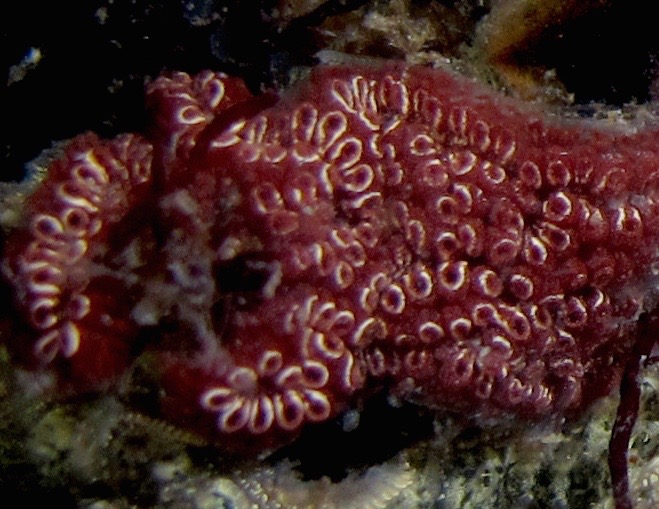


Figure S8. White on red color morphotype from Veracruz, Mexico. Photo by: H Bahena (ECOSUR, Mexico)

Table S1. Frequencies of COI haplotypes and ANT alleles at each sampled site for *Botrylloides nigrum*. For homozygous individuals at the ANT locus, allele frequencies were doubled to account for the presence of two identical alleles within an individual.

|  | **COI** | | | | | | | | |  | **ANT** | | | | | | | | | | | | | |
| --- | --- | --- | --- | --- | --- | --- | --- | --- | --- | --- | --- | --- | --- | --- | --- | --- | --- | --- | --- | --- | --- | --- | --- | --- |
| **Site** | **A** | **B** | **C** | **D** | **E** | **F** | **G** | **H** | **TOTAL** |  | **A** | **B** | **C** | **D** | **E** | **F** | **G** | **H** | **I** | **J** | **K** | **L** | **M** | **TOTAL** |
| **ATL** | 90 | 115 | 4 | 2 | 2 | 1 | 1 | 0 | 215 |  | 200 | 90 | 42 | 65 | 8 | 0 | 3 | 0 | 1 | 1 | 1 | 0 | 1 | 412 |
| **IR** | 11 | 35 | 0 | 0 | 0 | 0 | 0 | 0 | 46 |  | 51 | 16 | 10 | 7 | 0 | 0 | 3 | 0 | 0 | 0 | 1 | 0 | 0 | 88 |
| **TB** | 7 | 14 | 0 | 0 | 0 | 0 | 0 | 0 | 21 |  | 28 | 4 | 5 | 1 | 6 | 0 | 0 | 0 | 0 | 0 | 0 | 0 | 0 | 44 |
| **TX** | 6 | 7 | 0 | 0 | 0 | 0 | 0 | 0 | 13 |  | 8 | 9 | 1 | 8 | 0 | 0 | 0 | 0 | 0 | 0 | 0 | 0 | 0 | 26 |
| **VR** | 16 | 2 | 0 | 0 | 2 | 0 | 0 | 0 | 20 |  | 3 | 13 | 7 | 11 | 0 | 0 | 0 | 0 | 0 | 0 | 0 | 0 | 0 | 34 |
| **BA** | 0 | 1 | 0 | 0 | 0 | 0 | 0 | 0 | 1 |  | 2 | 0 | 0 | 0 | 0 | 0 | 0 | 0 | 0 | 0 | 0 | 0 | 0 | 2 |
| **BZ** | 1 | 13 | 0 | 0 | 0 | 1 | 0 | 0 | 15 |  | 25 | 5 | 0 | 0 | 0 | 0 | 0 | 0 | 0 | 0 | 0 | 0 | 0 | 30 |
| **PR** | 15 | 7 | 4 | 2 | 0 | 0 | 0 | 0 | 28 |  | 15 | 9 | 3 | 19 | 1 | 0 | 0 | 0 | 0 | 0 | 0 | 0 | 1 | 48 |
| **VZ** | 1 | 5 | 0 | 0 | 0 | 0 | 1 | 0 | 7 |  | 10 | 4 | 0 | 0 | 0 | 0 | 0 | 0 | 0 | 0 | 0 | 0 | 0 | 14 |
| **BOC** | 25 | 1 | 0 | 0 | 0 | 0 | 0 | 0 | 26 |  | 23 | 16 | 4 | 7 | 1 | 0 | 0 | 0 | 0 | 1 | 0 | 0 | 0 | 52 |
| **COL** | 3 | 0 | 0 | 0 | 0 | 0 | 0 | 0 | 3 |  | 5 | 0 | 1 | 0 | 0 | 0 | 0 | 0 | 0 | 0 | 0 | 0 | 0 | 6 |
| **BR** | 5 | 30 | 0 | 0 | 0 | 0 | 0 | 0 | 35 |  | 30 | 14 | 11 | 12 | 0 | 0 | 0 | 0 | 1 | 0 | 0 | 0 | 0 | 68 |
| **PAC** | 72 | 1 | 0 | 0 | 0 | 0 | 0 | 1 | 74 |  | 97 | 19 | 20 | 16 | 1 | 2 | 0 | 0 | 0 | 0 | 0 | 1 | 0 | 156 |
| **MAZ** | 15 | 0 | 0 | 0 | 0 | 0 | 0 | 0 | 15 |  | 20 | 1 | 6 | 3 | 0 | 0 | 0 | 0 | 0 | 0 | 0 | 0 | 0 | 30 |
| **PA** | 27 | 0 | 0 | 0 | 0 | 0 | 0 | 0 | 27 |  | 40 | 3 | 13 | 3 | 0 | 0 | 0 | 0 | 0 | 0 | 0 | 1 | 0 | 60 |
| **HI** | 29 | 1 | 0 | 0 | 0 | 0 | 0 | 1 | 31 |  | 37 | 14 | 0 | 10 | 1 | 2 | 0 | 0 | 0 | 0 | 0 | 0 | 0 | 64 |
| **SGP** | 1 | 0 | 0 | 0 | 0 | 0 | 0 | 0 | 1 |  | 0 | 1 | 1 | 0 | 0 | 0 | 0 | 0 | 0 | 0 | 0 | 0 | 0 | 2 |
| **IS/MED** | 24 | 8 | 0 | 0 | 0 | 0 | 0 | 0 | 32 |  | 22 | 15 | 23 | 3 | 0 | 0 | 0 | 3 | 0 | 0 | 0 | 0 | 0 | 66 |
| **TOTAL** | 186 | 124 | 4 | 2 | 2 | 1 | 1 | 1 | 321 |  | 319 | 124 | 85 | 84 | 9 | 2 | 3 | 3 | 1 | 1 | 1 | 1 | 1 | 634 |

Table S2. Chimeric individuals detected in this study, their location, their COI haplotypes, and the nucleotide position where a heteroplasmic mutation was detected. Asterisks represent new nucleotide diversity that was detected by resolving chimeric DNA.

| **Sample Code** | **Location (Ocean)** | **Haplotypes** | **Heteroplasmic Nucleotide Position** |
| --- | --- | --- | --- |
| HI19 | Oahu, HI, USA (PAC) | A + H* | 336 |
| VR05 | Veracruz, MX (ATL) | B + E* | 325 & 410 |
| VR07 | Veracruz, MX (ATL) | B + E* | 325 & 410 |
| PR05 | Puerto Rico (ATL) | B + C | 100 |
| PR11 | Puerto Rico (ATL) | C + D* | 100 |
| PR24 | Puerto Rico (ATL) | C + D* | 100 |
